# Less is more: wiring-economical modular networks support self-sustained firing-economical neural avalanches for efficient processing

**DOI:** 10.1093/nsr/nwab102

**Published:** 2021-06-10

**Authors:** Junhao Liang, Sheng-Jun Wang, Changsong Zhou

**Affiliations:** Department of Physics, Centre for Nonlinear Studies and Beijing-Hong Kong-Singapore Joint Centre for Nonlinear and Complex Systems (Hong Kong), Institute of Computational and Theoretical Studies, Hong Kong Baptist University, Hong Kong, China; Department of Physics, Shaanxi Normal University, Xi’An 710119, China; Department of Physics, Centre for Nonlinear Studies and Beijing-Hong Kong-Singapore Joint Centre for Nonlinear and Complex Systems (Hong Kong), Institute of Computational and Theoretical Studies, Hong Kong Baptist University, Hong Kong, China; Department of Physics, Zhejiang University, Hangzhou 310027, China; Beijing Computational Science Research Center, Beijing 100193, China

**Keywords:** neural network, cost efficiency, critical avalanche, modular network, mean-field theory

## Abstract

The brain network is notably cost-efficient, while the fundamental physical and dynamic mechanisms underlying its economical optimization in network structure and activity have not been determined. In this study, we investigate the intricate cost-efficient interplay between structure and dynamics in biologically plausible spatial modular neuronal network models. We observe that critical avalanche states from excitation-inhibition balance under modular network topology with less wiring cost can also achieve lower costs in firing but with strongly enhanced response sensitivity to stimuli. We derive mean-field equations that govern the macroscopic network dynamics through a novel approximate theory. The mechanism of low firing cost and stronger response in the form of critical avalanches is explained as a proximity to a Hopf bifurcation of the modules when increasing their connection density. Our work reveals the generic mechanism underlying the cost-efficient modular organization and critical dynamics widely observed in neural systems, providing insights into brain-inspired efficient computational designs.

## Teaser

This modelling, computational and theoretical study offers a fundamental understanding of the remarkable cost efficiency in neural systems that surprisingly achieves much more functional value with much less wiring and firing costs.

## INTRODUCTION

The interplay between the structure and dynamics of complex networked systems is a long-standing area of investigation, covering applications in complex systems from diverse scientific fields. Currently, research on this topic and applications in the brain and neuroscience are experiencing rapid growth.

Neurons in the human brain form a very huge and complex dynamic network for efficient functional processing with remarkable cost efficiency. The principles underlying its efficiency have been actively studied in recent years, either from a structural or dynamic aspect.

The brain network is very sparse globally: ∼100 billion neurons with ∼10^14^ synaptic connections each so that the overall density is ∼10^–8^ in the human brain [1]. However, the overall low-density connectivity is organized in a hierarchical manner from local circuits and cortical sheets to the whole-brain connectome [2,3]. Thus, a prominent feature of brain organization is that it is globally sparse with hierarchical, relatively dense, modular architectures [4–6], which is economical in network wiring since most of the connections are in the short range. There is ample evidence that brain networks can achieve local wiring cost minimization from the brain structure [7,8] and a trade-off between global wiring cost and processing efficiency [9,10].

Brain activities consume a low energy power of only *∼*20 W, which is remarkably energy efficient when compared to digital computers [11]. Dynamically, the irregular and sparse firing of neurons [12] can be collectively organized as oscillations and critical avalanches across different scales [13–16]. Such ‘scale-free’ dynamic activities were originally explained by critical branching theory [13], in which critical avalanches emerge near the transition point between a silent and an overactive phase. Later experimental evidence [14,17] supports that the transition point between an asynchronous and a synchronous phase better explains the observed critical avalanches, especially in terms of the existence of different critical exponents [14,18,19] satisfying scaling relations [20]. Functionally meaningful avalanche dynamics in critical synchronous transition states enable neurons to fire at a low rate [21]. Since cortical metabolic energy usage is dominated by action potentials and synaptic transmission [22–26], the avalanche dynamic is also energy economical to maintain the sustained spontaneous (resting) state, which consumes the majority of brain metabolic cost [27]. Finally, critical states are functionally beneficial by providing a broad dynamic range in response to stimulations [28,29] and thus a sensitive standby state for the brain to respond to constantly changing environments [30].

Although it is recognized that metabolic cost is a unifying principle governing neuronal biophysics [31], the fundamental mechanism underlying the economical interaction between structures and dynamic modes at the neural circuit level is not well understood. Specifically, how do the modular network (MN) structure and critical dynamics jointly achieve structural and dynamical optimization for energy-efficient processing? Deciphering these mechanisms is also important for developing brain-inspired efficient computing. Here, we address these questions with a biologically realistic neural dynamic model of excitation-inhibition (E–I)-balanced [32,33] spiking neuronal networks clustered in two-dimensional (2D) space to represent the resting-state dynamics on a cortical sheet composed of microcolumns. Interestingly, when rewiring the initial globally sparse random network (RN) into locally dense MNs, the firing rates decrease, the self-sustained dynamics change from asynchronous states to critical avalanche states, and the response sensitivity to weak transient external stimuli is greatly enhanced. Theoretically, we reveal the enhanced response of neurons by clustered firing and elucidate the dynamic transition via a Hopf bifurcation induced by denser connections within modules during rewiring through a novel mean-field theory. Overall, our integrative study of cost-structure-dynamics-function relationships in neural networks finds that locally dense connectivity under E–I-balanced dynamics appears to be the key ‘less-is-more’ solution to achieving cost-efficient organization.

## RESULTS

### Dynamic transition from RN to MN

**Figure 1.**
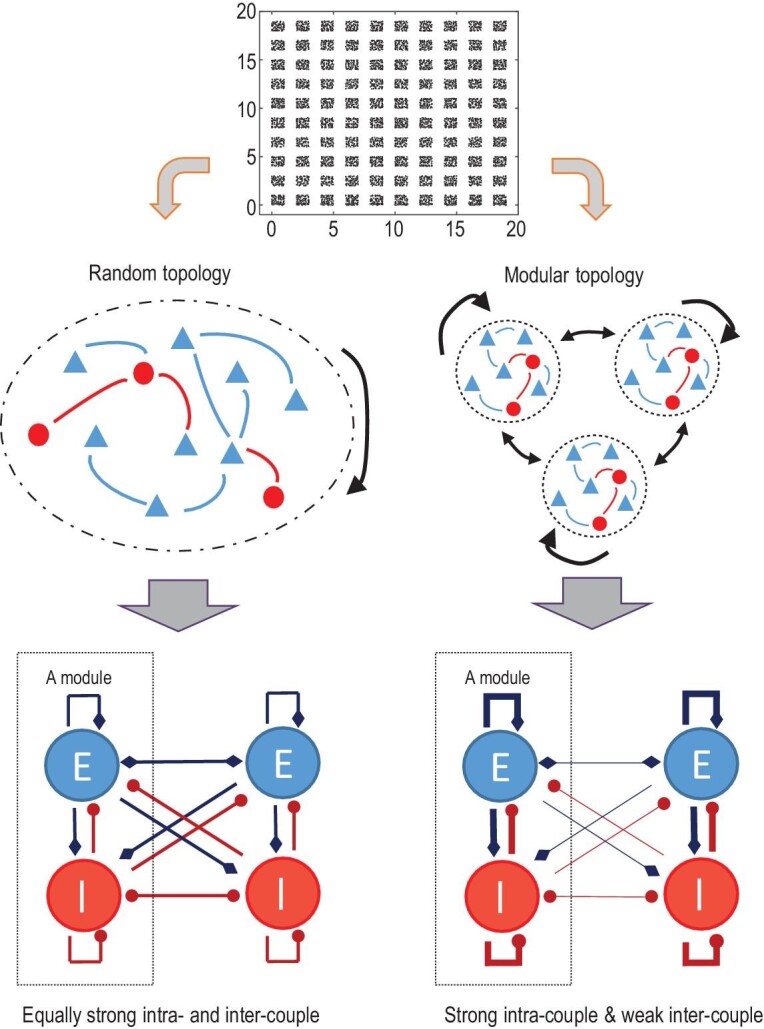
Diagram of the model neural network architecture. Neurons are placed in separated square modules in 2D space (top), mimicking the cortical sheet. Two network wiring patterns are distinguished: globally random topology (left) with equally dense intra- and inter-module coupling and modular topology (right) with dense intra-module coupling but sparse inter-module coupling. In this illustration of the model, the strength of coupling denotes the number of connections.

We study a model of $N\ = 5 \times {10^4}\ $neurons spread on a 2D plane. Considering that other tissues, such as vessels, may separate microcolumns of neurons, neurons are randomly placed on ${N_m} = \ 100$ square regions (modules). Modules are separated by blank space (Fig. 1 top), and each of the modules contains 500 neurons (80% excitatory and 20% inhibitory). Initially, we construct an RN by randomly connecting each neuron pair with a probability ${P_c}$ ( ${P_c} = \ 0.0017$ in the main text). To build an MN, inter-modular links are rewired, with a probability ${P_r}$, into the same module to become intra-modular links. The rewiring method is equivalent to constructing small-world networks, an essential feature of brain networks [34]. Here, the essential structural property captured in our model is that the network consists of coupled modules embedded in space [3,5,9]. The voltage (membrane potential) $V$ of a neuron in the E–I network is governed by conductance-based (COB) leaky integrate-and-fire (IF) dynamics [35],

(1) \begin{eqnarray*} \tau \ \frac{{dV}}{{dt}} &=& \left( {{V_{rest}} - V} \right)\ + {g_{ex}}\left( {V_E^{rev} - V} \right)\nonumber\\ &&+\, {g_{inh}}\left( {V_I^{rev} - V} \right)\!, \end{eqnarray*}

where $\tau ,{\rm{\ }}{V_{rest}},\ V_E^{rev},\ V_I^{rev}$ are the membrane time constant, resting (leaky) potential, and excitatory and inhibitory reversal potential, respectively. When a neuron receives a spike from an E, I neuron, its E, I conductance ${g_{ex}}$,$\ \ {g_{inh}}$ is changed as ${g_{ex}} \to {g_{ex}} + \Delta {g_e}$, ${g_{inh}} \to {g_{inh}} + \Delta {g_i}$, respectively, followed by exponential decay, $\tau _d^E \frac{{d{g_{ex}}}}{{dt}} = - {g_{ex}}$ and $\tau _d^I\ \frac{{d{g_{inh}}}}{{dt}} = \ - {g_{inh}}$. The network does not receive other external inputs. To launch the network activity, Gaussian white noise (GWN) is added to Equation (1) in the initial 200 ms and then removed. Then, we study the properties of its self-sustained dynamics without any external inputs. Details of the model parameters and simulation methods are provided in Supplementary Notes II. Neural Dynamics.

**Figure 2.**
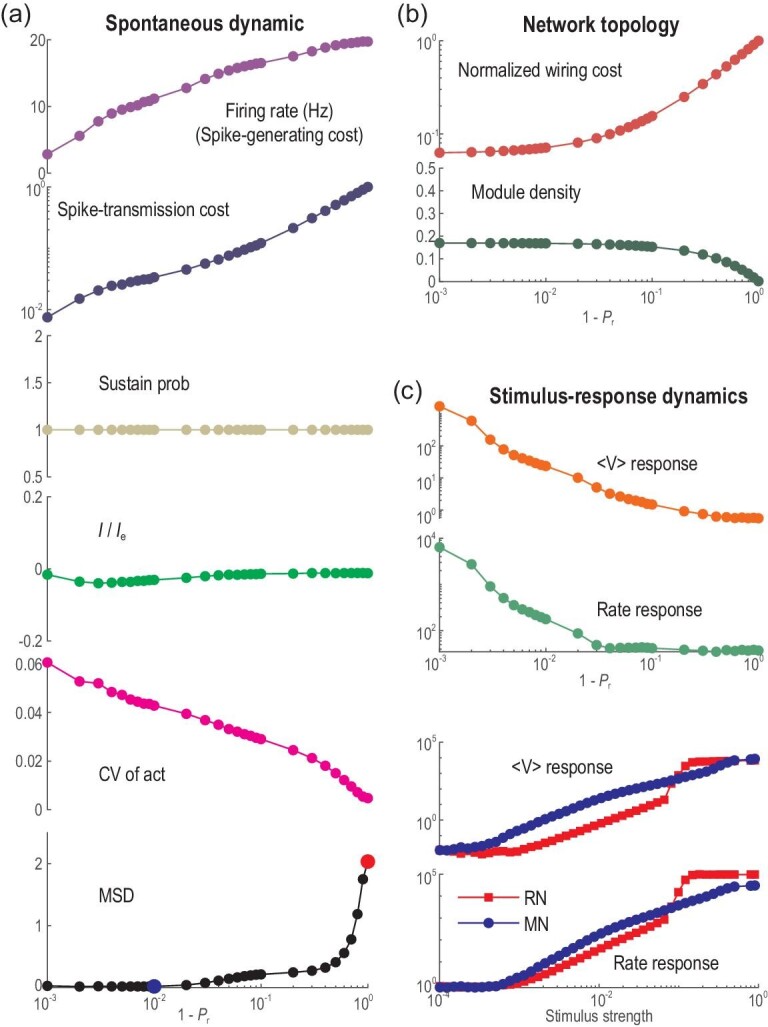
Wiring-economical modular networks support firing-economical avalanches and greatly enhance response sensitivity. (a) Spontaneous dynamic properties of the network during rewiring. From top to bottom: the average firing rate representing spike-generating cost; spike-transmission cost; self-sustained probability ${P_{sus}}$; current ratio $I/{I_e}$; CV of activity; and the MSD of avalanche distribution from power-law function. (b) Topological properties of the network during rewiring: normalized wiring cost of the whole network and connection density within a module. (c) Stimulus-response properties. The upper two panels show the response size of the membrane potential and firing rate during rewiring, where the stimulus strength is 1%. The lower two panels compare the responses of RN (${P_r} = \ 0$) and MN (${P_r} = \ 0.99$) under different stimulus strengths.

As the initial RN is rewired into the MN, it is interesting to find that the spike-generating and spike-transmission costs of the network are significantly decreased by orders of magnitude (Fig. 2(a)). Here, the spike-generating cost is defined as the average firing rate, and the spike-transmission cost of a neuron is defined as the product of its firing rate and the total length of its outgoing synapses (the average spike-transmission cost shown in Fig. 2(a) is normalized by the value of RN). These two measures for running costs mimic the energy cost for generating spikes and transmitting spikes [36,37]. In addition, since many links become local short-range connections, the normalized wiring cost (defined as the total Euclidian length of all links, normalized by the value of RN) is decreased by orders of magnitude, and the connection density within modules increases, approaching a value of ${p_c} \approx 0.17$ (${N_m}$ times the whole network density ${P_c}$, refer to Equation (4) below). Such smaller wiring length is desirable, as the reduced membrane areas of the fibres can reduce metabolic cost and reduce the transmission delay (although synaptic delay is not considered in our model for simplicity). The wiring cost reduction is more pronounced for larger networks with more modules (see Fig. S1 and detailed analysis in Supplementary Notes I. Network Setting). Thus, MN structures reduce both the wiring and running costs.

The initial large and sparse RN can self-sustain asynchronous activity without external input, giving a sustained probability $\ {P_{sus}} = \ 1.0$, which is maintained as the network is rewired into the MN (Fig. 1). This self-sustained activity [38] resembles the resting states of the brain and thus may play a functional role. The results are similar when the overall connection density ${P_c}$ changes (Fig. S4). However, a denser MN (larger ${P_c}$) with too weak inter-modular connections (${P_r} \to 1$) may not maintain self-sustained activities (Fig. S4). This breakdown of self-sustainability can be understood later by dynamic analysis of a separate module.

**Figure 3.**
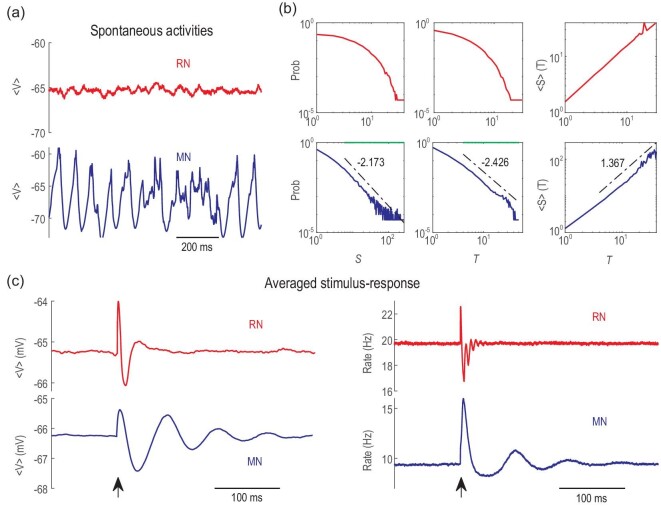
Dynamic comparison between RN (${p_r} = {\rm{\ }}0$) and MN (${p_r} = {\rm{\ }}0.995$). (a) Spontaneous activity (the average membrane potential) in a module. (b) The distributions of avalanche size $S$, avalanche duration $T$ and average size $\langle S\,\rangle$ given duration $T$. The upper/lower rows are the results for RN and MN, respectively. The green lines on top of the avalanche size and duration distribution under critical dynamics indicate the ranges of estimated power-law distributions. (c) Trial-averaged mean membrane potential (left) and mean firing rate (right) of a module, with transient stimuli (strength 1%) applied at the time marked by the arrows.

Interestingly, the dynamic modes of networks also covary during rewiring. RNs exhibit a classical E–I balanced asynchronous state with Poisson-like neuronal spiking [32]. We measure the balance by the net synaptic input current rescaled by the excitatory synaptic current ($I/{I_E}$) averaged over time and neurons. It maintains *∼*0 when an RN is changed into an MN (Fig. 2(a)), suggesting the maintenance of overall balance. The asynchronous state in the RN has a noisy fluctuation of the mean voltages around an equilibrium value (Fig. 3(a), upper panel). Spikes in the MN are clustered yet preserve irregular features and are interrupted by temporally silent periods (refer to Fig. S3(d) for raster plots of the spiking time in a module in an MN), exhibiting temporal dynamic variability in mean voltages (Fig. 3(a), lower panel), which can be measured from the CV (coefficient of variability, defined as standard deviation over absolute value of the mean) of the mean voltages of modules in each millisecond (Fig. 2(a)).

Importantly, MNs support critical neuronal avalanches in modules. Here, the time bin for measuring avalanches is the average inter-spike interval (ISI) of the merged spiking train [13] in a module. When rewiring an RN into a strong MN (e.g. ${P_r}$ = 0.995), the avalanche size and duration distribution of a module changes from exponential decay to a power-law (Fig. 3(b)). Statistical tests and estimations of critical exponents are made by an accustomed truncation algorithm [39]. Power-law avalanche size and duration distributions $P( S )\sim{S^{ - \tau }},$$P( T )\sim{T^{ - \alpha }}$ and $\langle S\,\rangle( T )\sim{T^{1/\sigma \upsilon z}}$ ($\tau \ = \ 2.173$, $\alpha \ = \ 2.426$, $\frac{1}{{\sigma \upsilon z}} = \ 1.367$ with $p$ value $ > 0.2$) are found in the truncated ranges, where scaling relation $\frac{{\alpha\, - 1}}{{\tau\, - 1}} = \frac{1}{{\sigma \upsilon z}}\ $[20] approximately holds (error ∼0.15). The size distribution is fitted into a power-law function [39], and its mean square deviation (MSD) from the fitted curve in Fig. 2(a) bottom shows that modules in an MN with ${P_r} \ge 0.99$ have avalanches with power-law distributions, exhibiting features of criticality. Other measurements of avalanches in MNs associated with the threshold of the average membrane potential are presented in Fig. S5, which also exhibits power-law distributions. This transition from asynchronous spiking to critical avalanche dynamics is the approach to a continuous synchronous transition point, as seen from the increase in the CV of activity (Fig. 2(a)). The self-sustained activity of coupled modules in the critical states provides the ideal scheme in which networks can work with a low firing rate. The reduced firing rate at criticality is a feature of the critical synchronous transition model [21], whereas the traditional branching process model does not exhibit this property—the firing rate of branching processes at critical states should be larger than that at subcritical states.

Critical states also induce greatly enhanced response sensitivity to transient stimuli. Here, the stimulus is modelled by raising the voltage $V$ to $ - 40$ mV of a proportion $x$ of the neurons in all modules. We call$\ x$ the stimulus strength. These neurons driven above the firing threshold emit spikes immediately, similar to optogenetic stimulation in experiments. The response sensitivity of a system can be reflected by the returning process of a signal to its baseline value after a transient perturbation. We measured the response in membrane potential and firing rate of the network modules. The size of the response is defined as $\mathop \smallint \nolimits_{{t_0}}^{{t_0} + T} | {f( t ) - {f_b}} |dt$, which is the area between the signal $f( t )$ (the trial-average voltage or firing rate of the network) and its resting value${\rm{\ }}{f_b}$, within a window of $T\ = \ 250\ {\rm{ms}}$, beginning from stimulus onset at ${t_0}$ (see also details in Supplementary Notes II. Neural Dynamics). As shown by the stimulus-induced trial-average voltage and firing rate of a module (Fig. 3(c)), the response of MNs is much larger and more pronounced than that of RNs. Interestingly, MNs show a stronger damped oscillation-like response pattern, which is a characteristic of event-related potentials in electroencephalogram signals of the brain's response to stimuli [40]. Importantly, both in membrane potential and in firing rate, MNs with critical avalanches exhibit response sensitivity that is much higher than RNs with asynchronous spiking activity for small stimulus strength (Fig. 2(c)). Furthermore, we check the dynamic range, defined as $\Delta {\rm{\ }} = {\rm{\ ln}}( {{x_{0.9}}/{x_{0.1}}} )$, where ${x_{0.9}},\ {x_{0.1}}$ are the stimulus strengths that induce 90% and 10% responses between the minimum and maximum values on a logarithmic scale, as in [28,29]. The dynamic ranges of MNs (${\Delta _V} = \ 6.28$,$\ {\Delta _r} = \ 5.47$) are greater than those of RNs (${\Delta _V} = \ 4.25$,$\ {\Delta _r} = \ 4.25$).

The above structure-dynamics relationships are robust with respect to the overall connection density ${P_c}$ (see Fig. S4) and hold in an extended modelling procedure where the number of inter-modular links decays with distance (see Fig. S6). In a word, MNs can support cost-efficient critical dynamical modes with greatly enhanced response sensitivity to encode variable input strength, whereas globally sparse RNs are both costly in architecture and in running and cannot properly respond to weak input signals.

### Structural correlation and dynamic transition of a single separate module

**Figure 4.**
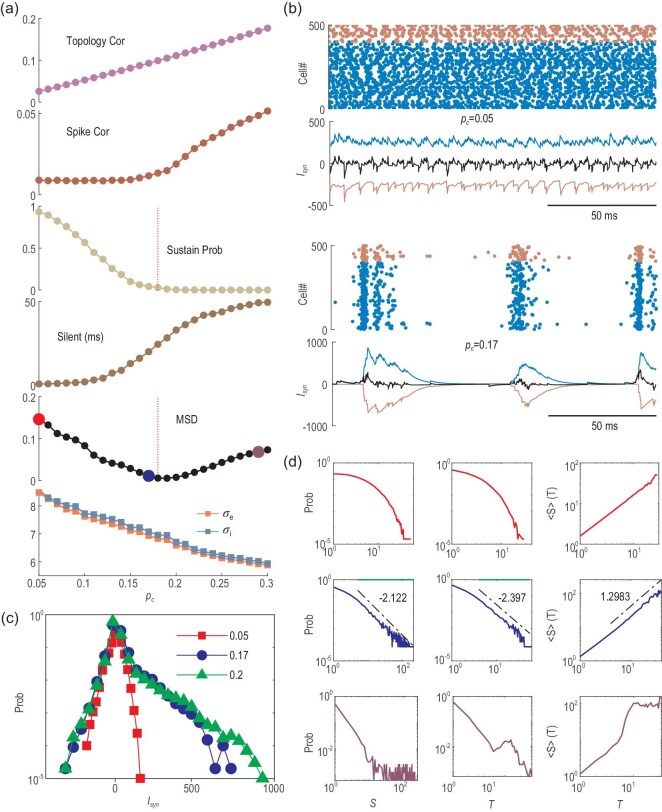
Spontaneous dynamic of a separate module. (a) The change in properties versus the density ${p_c}$. From top to bottom: topological correlation; spike correlation; sustained probability; averaged maximum silent period; the MSD of avalanche distribution from power-low fitting function and the numerically estimated effective parameters ${\sigma _E}$, ${\sigma _I}$ through Equation (3). (b) Raster plot of spiking time (blue, red for E, I cells) and synaptic currents (blue, red, black for E, I, net current) received by a neuron. The upper and lower panels are for ${p_c} = \ 0.05$ and $0.17$, respectively. (c) The distribution of net synaptic current received by a neuron for ${p_c}$= 0.05, 0.17 and 0.20. (d) Avalanche distributions (as Fig. 3(b)) for ${p_c} = \ 0.05$ (red), $0.17$ (blue) and $0.29$ (purple).

The key features in the structure-dynamics relationship can be understood from an isolated module separate from the whole network but subjected to a background excitatory Poisson input train with rate ${r_{in}}$. Here, we use ${r_{in}} = \ 50\ $Hz to approximate the weak input received by a neuron from other modules in the highly rewired region in the original network. As the rewiring probability ${P_r}$ increases, the local connection within a module becomes denser (Fig. 1 and Fig. 2(b)). For a separate module, as its connection density ${p_c}$ increases, neurons tend to have more common neighbours in the module, the common signal received by a pair of neurons becomes stronger, and their output spikes can be more correlated, as shown in Fig. 4(a).

This correlation in spiking changes the internal interactions in the network. Figure 4(b) shows the synaptic current of a randomly selected neuron. For low density, the net input current fluctuates slightly around zero due to strong E–I balance (Fig. 4(b), upper panels), and the distribution is close to a normal distribution (Fig. 4(c)). With higher density where spike correlation becomes prominent, correlated excitatory spikes induce quick activation of the network, followed by the activation of inhibitory neurons after an effective delay (due to slower inhibitory synaptic time), and then the activity is depressed. Thus, the net current exhibits oscillations around zero (thus, the network maintains the E–I balance on average), as shown in the lower panels of Fig. 4(b), and its distribution has a large tail on the positive side (Fig. 4(c)). The dynamic pattern is an alternation between synchronized firing and quiescent states with no spikes (Fig. 4(b), lower panels).

Furthermore, as the module becomes denser, the self-sustainability of the module decreases (Fig. 4(a)). Here, self-sustainability is tested by turning off the external inputs, i.e. letting ${r_{in}} = \ 0$ after the initial 200 ms. The activity almost cannot be sustained when ${p_c} > 0.17$, which is the module density in the original MN when ${P_r} \to 1$ (Fig. 2(b); see also Equation (4) below). The weaker sustainability of a denser network is a result of the clustered firing dynamic mode (Fig. 4(b), lower panel). The silent period during which no neuron fires increases with ${p_c}{\rm{\ }}$(Fig. 4(a)). If this period is too long, all recurrent inputs drop out, and the network activity dies out since there is no external driving.

Under fixed weak external background inputs ${r_{in}} = \ 50$ Hz, as the density ${p_c}$ increases, the network dynamics undergo a transition from asynchronous firing patterns (Fig. 4(b), upper panel) to critical avalanches (Fig. 4(b), lower panel) with reduced firing rates (Fig. 6(b)). The MSD of the avalanche size distribution from its best-fitted power-law function in Fig. 4(a) shows a minimum at *∼*${p_c} = \ 0.18$, close to the transition point of self-sustainability. Typical avalanche distributions for subcritical, critical and supercritical dynamic modes for ${p_c} = {\rm{\ }}0.05,{\rm{\ }}0.17$ and $0.29$ are shown in Fig. 4(d). Power-law avalanche size and duration distributions $P\!( S )\sim{S^{ - \tau }},$$P( T )\sim{T^{ - \alpha }}$ and $\langle S\,\rangle( T )\sim{T^{1/\sigma \upsilon z}}$ ($\tau \ = \ 2.122$, $\alpha \ = \ 2.397$, $\frac{1}{{\sigma \upsilon z}} = \ 1.298$ with $p$ value $ > 0.2$) are found for ${p_c} = {\rm{\ }}0.17$, where the scaling relation $\frac{{\alpha - 1}}{{\tau - 1}} = \frac{1}{{\sigma \upsilon z}}\ $[20] approximately holds (error ∼0.05).

Extended simulation of a separate module (Fig. S7) shows that this dynamic change is independent of input strength ${r_{in}}$, while the critical density ${p_c}$, where critical avalanches emerge, depends on ${r_{in}}$. Thus, the emergence of neuronal avalanches can be understood from the large transient fluctuations in the postsynaptic currents induced by correlation, leading to intermittent activities with lower rates.

### Effect of correlation: insight from a simplified model

**Figure 5.**
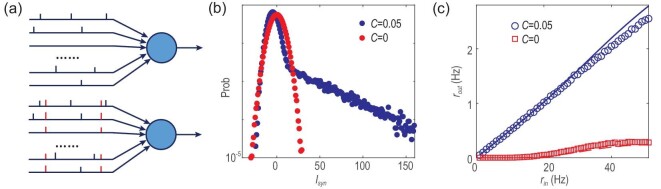
The simplified illustrative model. (a) The paradigm illustrating a neuron responding to uncorrelated (upper panel) or correlated (lower panel) random spike trains. A Poisson train (labelled in red) is copied into all input neurons to generate correlations among independent spike trains. (b) The distribution of the input signal when ${r_{in}} = \ 50\ $Hz when the common spike train is copied into a portion of randomly selected input synapses (see explanation in the text). (c) The response curve between input and output rate. Simulation results (symbols) are compared to theoretical predictions (curves). The red and blue colours are for uncorrelated ($C\ = {\rm{\ }}0$) and correlated ($C\ = {\rm{\ }}0.05$) input cases.

To quantitatively illustrate the impact of input correlation on response sensitivity under E–I-balanced dynamics, we can consider a simplified model as follows. A single neuron receives spike inputs from other *K = *200 Poisson excitatory spike trains. Each of the received spikes generates a unit of postsynapse current lasting for ${\tau _s} = \ 0.01\ {\rm{s}}$. The input signal of the neuron is the summation of these arriving spikes minus a constant equal to the mean current generated by these spikes to mimic the E–I balance. The input correlation is introduced by copying a common Poisson spike train into all input trains [41]; see Fig. 5(a) for a paradigm illustration. To construct spike trains with rate $r$ and correlation $C$, the common spike train has a rate $\alpha \ = \ Cr$, and independent spike trains have rates $\beta = ( {1 - C} ) r$. Assuming a threshold ($\theta \ = \ 20$) of the input signal above which the neuron fires a spike, we can numerically obtain the input-output rate response curves (Fig. 5(c)). Compared with independent input trains ($C\ = {\rm{\ }}0$), the correlation in inputs induces a positive tail in the distribution of the input signal (Fig. 5(b)), qualitatively capturing the feature of the IF module (Fig. 4(c)). In the simulation example of Fig. 5(b), the common spike train is copied into a portion of randomly selected input synapses at different times (such that the probability that more synapses receive spikes simultaneously is lower), resulting in a decaying positive tail in the distribution of the input signal when $C\ = {\rm{\ }}0.05$, which quantitatively resembles the observation from the spiking neural network simulations (Fig. 4(c)). We can see that the correlation increases the output rate when the input rate is the same (Fig. 5(c)). Moreover, this simplified model allows an analytic treatment to explain the effect of the correlation on the response rate (see Supplementary Notes III. Analysis of the Simplified Model with Correlated Inputs for details). The theoretical results (red dashed line for $C\ = {\rm{\ }}0$ and blue solid line for $C\ = {\rm{\ }}0.05$ in Fig. 5(c)) fit well to the simulation results of this simplified model.

Hence, the correlation in spikes injected from different recurrent synapses improves the responsiveness of neurons. With input correlation and response sensitivity, each neuron can maintain spike generation when the overall firing rate is low.

### Mean-field theory of single module dynamics

To further understand the dynamic mechanism underlying the transition of dynamic modes together with the reduction in firing rate, we derive the equations of average neural activity in each module and the interaction among the modules by a novel mean-field technique [19] (see Method and Supplementary Notes II. Neural Dynamics for details). The field equations of a single separate module with connection density ${p_c}$ receiving excitatory Poisson background input trains with rate ${r_{in}}$ are

**Figure 6.**
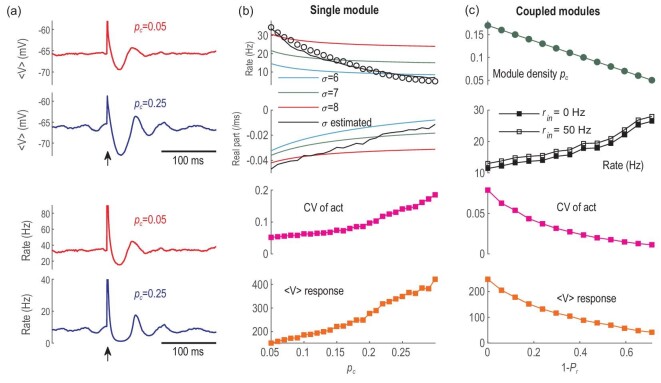
Mean-field theory to understand the dynamic transition. (a) Examples of the mean membrane potential and mean firing rate evolution of the single-module field equations when ${p_c} = \ 0.05$ and $0.25$, with transient stimuli applied at the time marked by the arrows. (b) Properties of the single-module field equations with different densities ${p_c}$. From top to bottom: the firing rate (circles are results from spiking network simulation, curves are results by fixing ${\sigma _\alpha } = \ 6,\ 7,\ 8$, and by ‘optimal’ ${\sigma _\alpha }$ given in Fig. 4(a)); real part of the eigenvalue of the fixed point; CV of activity; response size of membrane potential. (c) Properties of the coupled field equations with different rewiring probabilities ${P_r}$. From top to bottom: the corresponding density within a module; the firing rate; CV of activity; response size of membrane potential.

(2) \begin{equation*} \left\{ {\begin{array}{@{}*{1}{l}@{}} { \frac{{d{V_\alpha }}}{{dt}} = \frac{{{V_{rest}} - {V_\alpha }}}{\tau }}\\ {\qquad + \left[ {\tau _d^E{g_e}\!\left( {{r_{in}} + \sqrt {\frac{{{r_{in}}}}{{{N_\alpha }}}} \ {\xi _\alpha }\!\left( t \right)} \right) + {{\rm{\Phi }}_E}} \right]}\\ \qquad\quad \times \,{\left( {V_E^{rev} - {V_\alpha }} \right) + {{\rm{\Phi }}_I}\left( {V_I^{rev} - {V_\alpha }} \right))}\\ {\ \frac{{d{{\rm{\Phi }}_\alpha }}}{{dt}} = \ - \frac{{{{\rm{\Phi }}_\alpha }}}{{\tau _d^\alpha }} + {g_\alpha }{N_\alpha }{p_c}{Q_\alpha },\quad \alpha \ = \ E,I} \end{array}} \right., \end{equation*}

where ${V_E},\ {V_I}$ are the average E, I voltages, ${Q_\alpha } ( t ) = \ 1/[ {1 + {\rm{exp}}( {\frac{{{V_{th}} - {V_\alpha }}}{{{\sigma _\alpha }}}\frac{\pi }{{\sqrt 3 }}} )} ]$ is the average firing rate of $\alpha $ neurons, ${{\rm{\Phi }}_E},\ {{\rm{\Phi }}_I}$ are the average excitatory, inhibitory synaptic time courses received by the neuron and ${g_\alpha } = \frac{{\Delta {g_\alpha }}}{\tau }$. ${\xi _\alpha }$ are GWN terms. ${\sigma _\alpha }$ are effective parameters to construct the voltage-dependent mean population firing rate (see Method for more details). The strong complexity of COB IF dynamics challenges an analytical (self-consistent) estimation of the effective parameters ${\sigma _\alpha }$ [19]. Taking different fixed ${\sigma _\alpha }$, the field equations can qualitatively predict the decay of the rate with connection density ${p_c}$ (Fig. 6(b)). To achieve the best prediction, we numerically estimate the effective parameters ${\sigma _\alpha }$ through the formula

(3) \begin{equation*} {\sigma _\alpha } = \frac{{{V_{th}} - V_\alpha ^{ss}}}{{{\rm{ln}}\left( {{{\left( {Q_\alpha ^{ss}} \right)}^{ - 1}} - 1} \right)}}\ \frac{\pi }{{\sqrt 3}} \end{equation*}

from simulations of the single module IF spiking network. The simulations can numerically obtain the steady-state mean voltage $V_\alpha ^{ss} $ and mean firing rate $Q_\alpha ^{ss}$ of $\alpha $ neurons. The results of ${\sigma _\alpha }$ from modules with different densities ${p_c}$ are shown in the bottom panel of Fig. 4(a). Under this setting, the field equations can well quantitatively predict the decrease in firing rate as ${p_c}$ increases (Fig. 6(b)). Note that qualitative prediction can already be achieved by fixing the effective parameters ${\sigma _\alpha }$ value in Equation (2) (Fig. 6(b)). Importantly, the field equations reveal that the change in dynamics is associated with a (supercritical) Hopf bifurcation. The dominant eigenvalue of the equilibrium in Equation (2) is complex, and its real part approaches zero as ${p_c}$ increases (Fig. 6(b)). Thus, the firing rate oscillation emerges by approaching the Hopf bifurcation under noise perturbation, which induces critical avalanches [19]. However, the finite-size effect in a small module (500 neurons) hinders the precision of a mean-field theory. Thus, in the spiking IF model, the MSD achieves a minimum at *∼*${p_c} = \ 0.17$ (Fig. 4(a)), whereas the field equations do not reach the Hopf bifurcation point, and the dynamic is perturbed to bifurcation by noise. Note that a Hopf bifurcation indicates that a periodic motion emerges from zero amplitude, corresponding to the continuous increase of the synchrony in the spiking network (Fig. 4(a)). The CV of activity, measured by the firing rate series of field equations, grows as the ${p_c}$ increases (Fig. 6(b)). Finally, the response size of the voltage computed from the field equations (Fig. 6(b)) also qualitatively predicts the increase in response sensitivity for denser modules (examples of ${p_c} = {\rm{\ }}0.05$ and ${p_c} = {\rm{\ }}0.25$ are shown in Fig. 6(a), compared to Fig. 3(c)). This is because when approaching a bifurcation point, the system will respond more sensitively and will take a longer time to damp back to the fixed point after perturbation, a phenomenon called critical slowing down [42].

### Mean-field theory of the MN

The above investigation of separated modules with various connection densities under weak external background driving provides an understanding of the change in dynamic modes and firing rates with respect to the rewiring probability ${P_r}$ in the original MN (Fig. 1). First, there is a correspondence between the density in a module ${p_c}$ and the rewiring probability ${P_r}$:

(4) \begin{equation*} {p_c} = {P_c} \left( {1 + \left( {{N_m} - 1} \right)\!{P_r}} \right) \end{equation*}

(refer to Equation (S1.5)), as shown in Figs 6(c) and 2(b). Furthermore, the field equations of the whole MN can be written as (see Method and Supplementary Notes II. Neural Dynamics for details)

(5) \begin{eqnarray*} \left\{ \begin{array}{@{}*{1}{l}@{}} {\frac{{dV_\alpha ^k}}{{dt}} = \frac{{{V_{rest}} - V_\alpha ^k}}{\tau } + \left[ {\tau _d^E{g_e}\left( {{r_{in}} + \sqrt {\frac{{{r_{in}}}}{{{N_\alpha }}}} \ \xi _\alpha ^k\left( t \right)} \right) + {\rm{\Phi }}_E^k} \right]}\left( {V_E^{rev} - V_\alpha ^k} \right) + {\rm{\Phi }}_I^k\left( {V_I^{rev} - V_\alpha ^k} \right)\\ \frac{{d{\rm{\Phi }}_\alpha ^k}}{{dt}} = \ - \frac{{{\rm{\Phi }}_\alpha ^k}}{{\tau _d^\alpha }} + {g_\alpha }{N_\alpha }{P_c}[\left( {1 + \left( {{N_m} - 1} \right){P_r}} \right){Q_\alpha ^k + \mathop \sum \limits_{l \ne k} \left( {1 - {P_r}} \right)Q_\alpha ^l,\alpha \ = \ E,I,\ k\ = \ 1, \ldots ,{N_m}} \end{array} \right.,\nonumber\\ \end{eqnarray*}

with $V_\alpha ^k,\ {\rm{\Phi }}_\alpha ^k,\ Q_\alpha ^k,\ \xi _\alpha ^k$ corresponding to the quantities of $\alpha $ neurons in the *k*-th module (see Method for more details). Thus, the whole MN can be considered as ${N_m}$ coupled identical neural oscillators. During the rewiring process, the coupling strength between different modules (∼$1 - {P_r}$) decreases, whereas the self-coupling strength (∼$1 + ( {{N_m} - 1} ){P_r}$) increases. In this process, although different modules become less affected by each other, the increase in their internal density significantly shapes the dynamic properties of each module, as revealed in separated modules (Fig. 4). Here, the effective parameters ${\sigma _E},\ {\sigma _I}$ to construct $Q_\alpha ^k$ in Equation (5) depend on the rewiring probability ${P_r}$ through their optimal dependence on ${p_c}$ in separated modules shown in the bottom panel of Fig. 4(a) and the relationship between ${P_r}$ and ${p_c}$ (Equation (4)). The numerical results in Fig. 6(c) show that the coupled field equations qualitatively predict the decrease in firing rate and increase in CV of activity and response sensitivity to transient stimuli for increasing rewiring probability ${P_r}$, as observed in the spiking neural model in Fig. 2. Furthermore, Equation (5) with ${r_{in}} = \ 0$ also predicts a decrease in the nonzero firing rate (Fig. 6(c)), which is a qualitative prediction of self-sustainability during rewiring (Fig. 2(a)). Note, however, that the mean-field analysis here does not capture the effect of changes in input patterns (e.g. increased input correlation) of a module during the rewiring process of the MN. This is a source of prediction errors that lead to the difference between single-module field equations and a module in the coupled-modules field equations when the latter is constructed by the ${\sigma _\alpha }$ parameters of the former (refer to Fig. S8). An improvement in the future may be made by assuming oscillatory input in the coupled-modules field model where the oscillatory amplitude increases with rewiring. To conclude, the mean-field theory predicts the dynamical transition (approaching a Hopf bifurcation) of a module with increasing internal density, and this emergent behaviour is maintained for the whole MN with mutually coupled modules when rewiring the inter-modular links to intra-modular links.

## CONCLUSION AND DISCUSSION

In this study, we have unveiled the principle of neural networks allowing cost-efficient optimization in both structure and dynamics simultaneously. There have been many studies on either side, considering the optimization of brain network structure [7–9] or energy-efficient neural dynamics [22–26]. For example, energy-efficient cortical action potentials are facilitated by body temperature [25], and cellular ion channel expression is optimized to achieve function while minimizing the metabolic cost of action potentials [31]. However, most previous studies considered the efficiency of the network structure (wiring cost) or the efficiency of dynamics (running cost) separately. Here, considering both structure and dynamics at the circuit level, we show that a wiring-economical MN can support response-sensitive critical dynamics with a much lower running cost while maintaining self-sustainability. This is a notable counterintuitive ‘less-is-more’ result because we obtained greatly enhanced functional values with significant decreases in cost rather than a trade-off between them.

In our model, the efficiency of activity is achieved by critical avalanche states. Different from the traditional critical branching region, the critical dynamics in the synchronous transition region simultaneously achieve greater response sensitivity and a lower firing rate. Previous studies have shown that critical avalanches can appear under various network topologies, for example, scale-free networks with small-world features [43]. Here, we show that a locally dense but globally sparse MN is an efficient organization of the network structure that enables both a low global wiring cost and response-sensitive critical dynamics with a low running cost. It would be interesting to further explore its dynamic advantages on specific cognitive tasks such as working memory recall and decision making.

The origin and mechanism of functionally sensitive critical dynamics in neural systems [13–16,28–30] is a long-standing, challenging and controversial topic. Considering the physical mechanism that supports such a co-optimization of structure and dynamics, here we reveal that with increasing topological correlation in the E–I balanced network, the spike correlation increases, and so does the fluctuation of the inputs received by neurons. In this case, neurons can be activated by a lower firing rate, and the network has higher sensitivity. From the perspective of nonlinear dynamics, these features are captured by a novel mean-field analysis, which reduces the whole MN into coupled oscillators describing the macroscopic dynamics of each module. We elucidate the dynamic mechanism for producing avalanches in proximity to a Hopf bifurcation in the mean field. Close to the bifurcation point, the resulting synchronized spikes in each module are temporally organized as critical avalanches. This stronger collective firing rate variability allows greater computation and coding power [44]. In the highly (yet not totally) rewired MN, the sparse inter-modular connections can provide weak external input to a module from other modules. Meanwhile, as modules are dense enough to be around the response-sensitive critical dynamic states, these weak inputs are sufficient to maintain the whole MN in self-sustained states with low rates.

In principle, the analytical theory for treating biologically plausible COB IF neuronal networks is still an open question [45]. Our approximation semi-analytical mean-field technique serves as an effective theory to study the macroscopic dynamics of such realistic networks. It is important to stress that our work put several important features of neural systems into an integrated framework. Spatial embedding of neural circuits under the wiring cost constraint gives rise to local dense connections and modular organization [7–9]. The E–I balance is a fundamental property of neural circuits [32,33]. Collective activities such as critical avalanches and oscillations are pronounced dynamic features of neural networks [13–16,28–30]. Our modelling and theoretical analysis framework reveals intricate interactions among wiring and running costs, MN topology, critical avalanche dynamic modes and sensitive responses to weak stimulations. Thus, it provides an integrative principle for structural-dynamic cost-efficient optimization in neural systems. Our integrative studies with generic network manipulation and novel mean-field theory with realistic neural dynamics can be extended to coupled cortical areas to offer an understanding of critical dynamics across the whole brain [46,47] based on a hierarchical modular connectome [48]. Furthermore, spatial networks with connections to only the nearest neighbours can exhibit propagating waves with critical dynamic properties [49]. It would be interesting to generalize our model to such nearest-neighbour coupling scenarios and explore the effect of extrasparse long-range connections in such models. This type of model may share similar principles revealed in this study, as in our extended model where short-distance links are dominant (see Fig. S6). The physical principles revealed in our work can guide further development of brain-inspired efficient computing [50].

## METHOD

### Mean-field theory of IF neural dynamics

In this section, we present the outline of the mean-field theory for deriving the field equations Equation (2) and Equation (5). More details are provided in Supplementary Notes II. Neural Dynamics. In our model, for the *i*-th neuron in the *k*-th module, we denote its spiking train as $\{ {t_i^k( n ),\ n \ge 1} \}$, its $\alpha $ (E or I) neighbours in the *l*-th module as $\partial _{k, i}^{l,\alpha }$, its voltage as $V_i^k( t )$, its input conductance received from recurrent excitatory, recurrent inhibitory neurons as $GE_i^k( t ),\ GI_i^k( t )$, its external input spike trains (with rate ${r_{in}}$) and input conductance from external neurons as $\{ {T_i^k( n ),\ n \ge 1} \}$ and $GO_i^k( t )$ (if there are external inputs). Then, the network dynamic equation Equation (1) can be written in the following more specific form:

(6) $$\begin{equation*}\left\{{\begin{array}{@{}*{1}{l}@{}} {\frac{{dV_i^k}}{{dt}} = \frac{{{V_{rest}} - V_i^k}}{\tau }\ + \left[ {GE_i^k\left( t \right) + GO_i^k\left( t \right)} \right]\left( {V_E^{rev} - V_i^k} \right) + GI_i^k\left( t \right)\left( {V_I^{rev} - V_i^k} \right)}\\ {\frac{{dGE_i^k\left( t \right)}}{{dt}} = \ - \frac{{GE_i^k\left( t \right)}}{{\tau _d^E}} + {g_e}\left[ {\sum\nolimits_{j \in \partial _{k,i}^{k,E}} {\sum\nolimits_n \delta } \left( {t - t_i^k\left( n \right)} \right) + \sum\nolimits_{l \ne k} {\sum\limits_{j \in \partial _{k,i}^{l,E}} {\sum\nolimits_n \delta } } \left( {t - t_i^l\left( n \right)} \right)} \right]}\\ {\frac{{dGI_i^k\left( t \right)}}{{dt}} = \ - \frac{{GI_i^k\left( t \right)}}{{\tau _d^I}} + {g_i}\left[ {\sum\nolimits_{j \in \partial _{k,i}^{k,I}} {\sum\nolimits_n \delta } \left( {t - t_j^k\left( n \right)} \right) + \sum\nolimits_{l \ne k} {\sum\limits_{j \in \partial _{k,i}^{l,I}} {\sum\nolimits_n \delta } } \left( {t - t_i^l\left( n \right)} \right)} \right]}\\ {\frac{{dGO_i^k\left( t \right)}}{{dt}} = \ - \frac{{GO_i^k\left( t \right)}}{{\tau _d^E}} + {g_e}\sum\nolimits_n \delta \left( {t - T_i^k\left( n \right)} \right)} \end{array}} \right.,
\end{equation*}$$

where ${g_e} = \frac{{\Delta {g_e}}}{\tau }\ ,\ {g_i} = \frac{{\Delta {g_i}}}{\tau }\ $.

Denote $V_{E}^{k} = {\langle V_{i}^{k}}\rangle_{i \in k,E}$, $V_{I}^{k} = {\langle V_{i}^{k}}\rangle_{i \in k,I}$, ${\rm{\Phi }}_{E}^{k} = \langle GE_{i}^{k}\rangle_{i \in k,Eork,I}\ $and ${\rm{\Phi }}_{I}^{k} = {\langle GI_{i}^{k}}\rangle_{i \in k,Eork,I}$. We first adopt a diffusion approximation that $GO_{i}^{k}( t ) \approx \tau _{d}^{E}{g_{e}}( {{r_{in}} + \sqrt {{r_{in}}} \xi _{i}^{k}( t )} )$, with ${\{ {{\rm{\xi }}_{i}^{k}( t )} \}_{k,j}}$being independent standard GWNs. Thus, $\langle GO_{i}^{k} ( t )\rangle_{i \in k,\alpha } \approx \tau _d^E{g_e}( {{r_{in}} + \sqrt {\frac{{{r_{in}}}}{{{N_{\alpha} }}}} \xi _{\alpha} ^{k}( t )} )$, with ${\{ {\xi _{\alpha} ^{k}( t )} \}_{k,\alpha }}$being independent standard GWNs. Then, taking the average $\langle\,\rangle_{i \in k,\alpha }$ to the first equation of Equation (6), with the decoupling approximation $[ \langle{GE_{i}^{k} + GI_{i}^{k}} ]{V_{i}^{k}}\rangle_{i \in k,\alpha } \approx \langle{GE_{i}^{k}} + {GI_{i}^{k}}\rangle_{i \in k,\alpha }\langle{V_{i}^{k}}\rangle_{{i \in k,\alpha }}$, we obtain the first equation of Equation (5). Next, the firing rate of the $\alpha $ neurons in the *k*-th module can be approximated as $Q_{\alpha} ^{k}( t ) = \langle\mathop \sum \nolimits_{n} \delta {( {t - t_j^k( n )} )\rangle_{j \in k,\alpha }} = 1/[ {1 + {\rm{exp}}( {\frac{{{V_{th}} - V_{\alpha} ^{k}}}{{{\sigma _{\alpha} }}}\frac{\pi }{{\sqrt 3 }}} )} ]$ [19]. This form essentially captures the sub- and supra-threshold microscopic dynamics of a spiking network, that is, $Q_\alpha ^k( t )$ represents the proportion of $\alpha $ type neurons that spike between $t$ and $t + \Delta t$ ($\Delta t$ is an infinitely small quantity) as well as the mean firing rate of $\alpha $ type neurons at time $t$ with unit per ms. Here, ${\sigma _\alpha }$ are effective parameters to construct the voltage-dependent mean population firing rate. Note that this approximation scheme based only on the first-order statistics neglects several factors that affect the accurate firing rate, including higher-order statistics, noise correlation and refractory time. Thus, it does not have an analytical form, and ${\sigma _\alpha }$ should be estimated numerically.

Under the mean-field approximation, we have $\mathop \langle\sum \nolimits_{j \in \partial _{k, i}^{l, \alpha }} \mathop \sum \nolimits_n \delta {( {t - t_j^l( n )} )\rangle _{i \in k, E\ or\ k, I}} = n_\alpha ^{kl} Q_\alpha ^l( t )$, where $n_\alpha ^{kl}$ is the average number of $\alpha $ neighbours in the *l*-th module of a neuron in the *k*-th module. Thus, $n_\alpha ^{kl} = {p^{kl}}\ {N_\alpha }$, where ${p^{kl}}$ is the connection probability from module $l$ to module $k$ so that

(7) \begin{equation*} {p^{kl}} = \left\{{\begin{array}{@{}*{2}{l}@{}} {P_{intra}} = {P_c}\left( {1 + \left( {{N_m} - 1} \right){P_r}} \right),&\,\, k = l\\ {P_{inter}} = {P_c}\left( {1 - {P_r}} \right),&\,\, k \ne l \end{array}} \right.\,. \end{equation*}

Taking ${\langle\,\,\rangle }_{i \in k, E}$ or ${\langle\,\,\rangle } _{i \in k, I}$ to the second and third equations of Equation (6), we get the second equation of Equation (5), which finishes the construction of the coupled field equations.

In the limit of ${P_r} \to 1$ (all rewired), modules are almost separated. Let ${P_r} = \ 1$ in Equation (5), and we obtain the field equations corresponding to one separate module with additional external excitatory inputs, i.e. Equation (2), with ${p_c} = {P_c} {N_m}$ being the connection density of the module.

## Supplementary Material

nwab102_Supplemental_File
