# Supplementary material for: Less is more: wiring-economical modular networks support self-sustained firing-economical neural avalanches for efficient processing
Source: Natl Sci Rev. 2021 Jun 10;9(3):nwab102. doi: 10.1093/nsr/nwab102 (PMC8962757; doi:10.1093/nsr/nwab102)
Supplement: nwab102_Supplemental_File [file nwab102_supplemental_file.docx]

Supplementary Materials:

Less is More: Wiring-Economical Modular Networks Support Self-Sustained Firing-Economical Neural Avalanches for Efficient Processing

Junhao Liang, Sheng-Jun Wang, and Changsong Zhou

**This file includes:**

Supplementary Notes I. Network Setting

Supplementary Notes II. Neural Dynamics

Supplementary Notes III. Analysis of the Simplified Model with Correlated Inputs

Supplementary references

Figures. S1 to S8

**Supplementary Notes I. Network Setting**

The whole system consists of $N$ neurons, 80% are excitatory neurons and 20% are inhibitory neurons. To model a local cortical surface, neurons are placed on square regions which are separated by blank space on a two-dimensional (2D) plane, as illustrated in Fig. 1 top panel in the main text. To measure the distance between different neurons, we first assume the length of each square and the width of the blank space are set as 1 (Fig. 1 top panel). Each neuron $i$, with the coordinate of its position $(x_{i}, y_{i})$, is randomly distributed in each square. In all cases, we assume each module (square) consists of $S_{m}=500$ neurons and the ratio between excitatory and inhibitory neurons in each module is kept as 4:1. The example in Fig. 1 top panel contains $N_{m}=10\times10$ modules and in this case the whole network size is $N=N_{m}\times S_{m}=50,000$. Throughout the work, the default setting is $N_{m}=100$, $S_{m}=500$ so that $N=50,000$ and $P_{c}=0.0017$ unless extra specifying.

The random network (RN) is built by connecting each possible neuron pair with a probability $P_{c}$. To build a modular network (MN), links between modules are rewired, with a probability $P_{r}$, into square to become intra-modular links. For example, for an inter-module link $i\to j$ whose source neuron $i$ and target neuron $j$ are in different square modules, we replace the target node $j$ with a randomly selected neuron $k$ in the same module of $i$ (initially a link between $i$ and $k$ is absent). This rewiring makes the connection probability in a module higher than that between different modules, forming modular structure in the network while maintaining connection density of the whole network $P_{c}$ unchanged, that is, the total number of links on average is $M={{NP}_{c}(N-1)\approx N^{2}P}_{c}$, which is fixed during the rewiring process.

In the initial random network, the number of intra-module links, denoted by $M_{intra}$, is

$M_{intra}=S_{m}\left( S_{m}-1 \right)P_{c}N_{m}\approx NS_{m}P_{c}.$ (S1.1)

The number of inter-modular links in RN, denoted by $M_{inter}$, is

$M_{inter}=\left( N_{m}-1 \right)NS_{m}P_{c}$. (S1.2)

In this case, both the intra-module and inter-module connection densities are $P_{c}$, that is,

$P_{intra}=P_{inter}=P_{c}$. (S1.3)

In the MN with the rewiring probability $P_{r}$, the number of intra-modular links becomes

$M_{intra}^{'}=M_{intra}+M_{inter}P_{r}=[1+(N_{m}-1)P_{r}]NS_{m}P_{c}.$ (S1.4)

Thus the intra-module density is

$P_{intra}=\frac{M_{intra}^{'}}{N(S_{m}-1)}\approx\frac{M_{intra}^{'}}{NS_{m}}=P_{c}(1+(N_{m}-1)P_{r})$. (S1.5)

The number of inter-modular links in MN becomes

$M_{inter}^{'}=M_{inter}\left( 1-P_{r} \right)=\left( N_{m}-1 \right)NS_{m}P_{c}\left( 1-P_{r} \right)$. (S1.6)

Thus the inter-module density is

$P_{inter}=\frac{M_{inter}^{'}}{\left( N_{m}-1 \right)NS_{m}}{=P}_{c}(1-P_{r})$. (S1.7)

The length of a link from neuron $i$ to neuron $j$ is the Euclidean distance between the pair of neurons in the 2D plane $l_{ij}=\sqrt{\left( x_{i}-x_{j} \right)^{2}+\left( y_{i}-y_{j} \right)^{2}}$. The normalized wiring cost of the whole network is defined as the summation of the length of all links rescaled by that value in initial RN without rewiring. Thus, the normalized wiring cost of initial RN is 1.

Fig. S1 shows the relation between the normalized wiring cost and the rewiring probability $P_{r}$. As the rewiring probability $P_{r}$ increases, the wiring cost decreases. When the value of $P_{r}$ is larger than 0.99, the normalized wiring cost tends to a constant. The results also hold for different module numbers (Fig. S1). This normalized wiring cost in MN is independent of the overall connection density $P_{c}$, as shown in Fig. S4 bottom panels where the costs with connection density $P_{c}=0.001$, $0.0015$,$0.002$ are presented. This property can be understood by an analytic treatment as follows. We assume that the mean length of the intra-modular links is $l$, whereas the mean length of the inter-modular links is $L$. The normalized wiring cost is

$cost=\frac{M_{intra}^{'}l+M_{inter}'L}{M_{intra}l+M_{inter}L}=\frac{\left( S_{m}-1 \right)l+S_{m}\left( N_{m}-1 \right)P_{r}l+S_{m}\left( N_{m}-1 \right)\left( 1-P_{r} \right)L}{\left( S_{m}-1 \right)l+S_{m}\left( N_{m}-1 \right)L}$. (S1.8)

Thus, it is independent of the connection density $P_{c}$ of the network, the same as the results shown in Fig. S4 bottom panels. As the rewiring probability $P_{r}$ tends to 1.0, the normalized wiring cost is

$cost=\frac{\left( S_{m}-1 \right)l+S_{m}\left( N_{m}-1 \right)l}{\left( S_{m}-1 \right)l+S_{m}\left( N_{m}-1 \right)L}\approx\frac{l}{L}$. (S1.9)

Furthermore, the mean intra-modular link length $l$ does not change with the size of the 2D plane, while the mean inter-modular link length $L$ increases with the size of plane. Therefore, the normalized wiring cost decreases with the size of the system, as shown in Fig. S1.


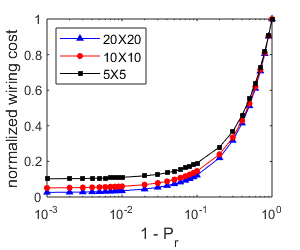


Fig. S1. The normalized wiring cost (rescaled by the initial RN) of MN versus the rewiring probability $P_{r}$. The number of modules are $N_{m}=5\times5, 10\times10$ and $20\times20$ respectively for $P_{c}=0.0017$.

**Supplementary Notes II. Neural Dynamics**

**2.1 Spiking dynamics of neurons**

In our model, each module has $N_{E}=400$ excitatory (E) neurons and $N_{I}=100$ inhibitory (I) neurons (totally 500 in a module). For each module, labels 1~400 are E neurons and labels 401~500 are I neurons. For the *i*-th neuron in *k*-th module, we denote its spiking train as $\{t_{i}^{k}\left( n \right), n\geq1\}$, its $\alpha$ (E or I) neighbors in the *l*-th module as $\partial_{k,i}^{l,\alpha}$, its voltage as $V_{i}^{k}(t)$, its input conductance received from recurrent excitatory, recurrent inhibitory neurons as ${GE}_{i}^{k}\left( t \right),{GI}_{i}^{k}\left( t \right)$, its external input spike trains (with rate $r_{in}$) and input conductance from external as $\{T_{i}^{k}\left( n \right), n\geq1\}$ and ${GO}_{i}^{k}\left( t \right)$ (if there are external inputs). Thus, they obey the equations

$\left\{ \begin{aligned} \frac{dV_{i}^{k}}{dt}=\frac{V_{rest}-V_{i}^{k}}{\tau}+\left[ {GE}_{i}^{k}\left( t \right)+{GO}_{i}^{k}\left( t \right) \right]\left( V_{E}^{rev}-V_{i}^{k} \right)+{GI}_{i}^{k}\left( t \right)\left( V_{I}^{rev}-V_{i}^{k} \right) \\ \frac{d{GE}_{i}^{k}\left( t \right)}{dt}=-\frac{{GE}_{i}^{k}\left( t \right)}{\tau_{d}^{E}}+g_{e}[\sum_{j\in\partial_{k,i}^{k,E}} \sum_{n} \delta\left( t-t_{i}^{k}\left( n \right) \right)+\sum_{l\neq k} \sum_{j\in\partial_{k,i}^{l,E}} \sum_{n} \delta\left( t-t_{i}^{l}\left( n \right) \right)] \\ \frac{d{GI}_{i}^{k}\left( t \right)}{dt}=-\frac{{GI}_{i}^{k}\left( t \right)}{\tau_{d}^{I}}+g_{i}[\sum_{j\in\partial_{k,i}^{k,I}} \sum_{n} \delta\left( t-t_{j}^{k}\left( n \right) \right)+\sum_{l\neq k} \sum_{j\in\partial_{k,i}^{l,I}} \sum_{n} \delta\left( t-t_{i}^{l}\left( n \right) \right)] \\ \frac{d{GO}_{i}^{k}\left( t \right)}{dt}=-\frac{{GO}_{i}^{k}\left( t \right)}{\tau_{d}^{E}}+g_{e}\sum_{n} \delta(t-T_{i}^{k}\left( n \right)) \end{aligned} \right.$ , (S2.1)

where $g_{e}=\frac{\Delta g_{e}}{\tau}, g_{i}=\frac{\Delta g_{i}}{\tau}$. Parameters in simulation are [1]: $\tau=20ms$*, $\tau_{d}^{E}=5ms$,$\tau_{d}^{I}=10ms$ ,$V_{rest}=-60mV$,$V_{E}^{rev}=0mV$,$V_{I}^{rev}=-80mV$,$\Delta g_{e}=0.5$,* $\Delta g_{i}=5$. $\{T_{i}^{n}\}$ is the Poisson spiking trains injected to the *i*-th neuron from external whose rate is $r_{in}$ Hz. Spiking reset threshold is $V_{th}=-50mV$. In simulation, we also apply a refractory period 5ms.

In the original networks with 100 modules, there is no external input, i.e. ${GO}_{i}^{k}\left( t \right)=0$. To launch the network activity, a Gaussian white noise (GWN) term, $\xi_{i}^{k}(t)$, is added to the first equation of Eq. (S2.1) in the initial 200ms and then removed. It satisfies $\left\langle\xi_{i}^{k}(t) \right\rangle=0$ and $\left\langle\xi_{i}^{k}(t)\xi_{i}^{k}(s) \right\rangle=\tau^{2}D\delta(t-s)$. $\xi_{i}^{k}$ are independent of each other for different $k,i$. Here, we use noise strength $D=10$. The properties of the self-sustained dynamics is independent of this noise strength.

In the model of a separate module simulation, there is an external input spike train $\{T_{i}^{k}\left( n \right), n\geq1\}$ with rate $r_{in}=50Hz$.

We use 100 realizations of the network in computing the probability of sustained activity $P_{sus}$. If the network still spikes at 1 second after the noise or external input has been removed, it is regarded to exhibit self-sustained activity.

**2.2 Stimulus-response relation**

**2.2.1 Measure the response sensitivity in spiking networks**

In experimental studies on the response of the brain to the cognitive events, it is necessary to average the measured brain activity over trails of experiments. Here, the stimulus method is that $x$ proportion of the randomly selected neurons in all modules are activated by increasing their membrane potential to $-40mV$ (above the threshold) in one simulation step. $x$ is termed the stimulus strength in our study.

We measure the response sensitivity in terms of the membrane potential properties and the spiking properties, using the average membrane potential and firing rate of the network modules. To study the sensitivity, we can consider the returning process of a signal to its baseline value after a transient perturbation.

The collective behavior of a module $k$ responding to additional stimulus can be reflected by its mean membrane potential and its mean firing rate: $\left\langle V^{k}\left( t \right) \right\rangle=\frac{1}{s_{m}}\sum_{i=1}^{s_{m}} V_{i}^{k}\left( t \right)$, $\left\langle Q^{k}\left( t \right) \right\rangle=\frac{1}{s_{m}}\sum_{i=1}^{s_{m}} Q_{i}^{k}\left( t \right)$ (where $Q_{i}^{k}\left( t \right)$ is the neuronal firing rate series constructed in 1ms). Averaged signals $\left\langle V\left( t \right) \right\rangle=\frac{1}{N_{m}}\sum_{k=1}^{N_{m}} \left\langle V^{k}\left( t \right) \right\rangle$,$\left\langle Q\left( t \right) \right\rangle=\frac{1}{N_{m}}\sum_{k=1}^{N_{m}} \left\langle Q^{k}\left( t \right) \right\rangle$ (averaged over all the modules) are then used as the measured signal $f(t)$. We measure the response size by $\int_{t_{0}}^{t_{0}+T} |f\left( t \right)-f_{b}|dt$, the area of the region (colored region in Fig. S2) between the signal curve $f\left( t \right)$ and the baseline of the ongoing activity $f_{b}$. The length for recording the response is $T=250ms$ after the stimulus onset at $t=t_{0}$. Results are averaged over 100 realizations of the simulation.

An example of the averaged signal is shown in Fig. S2. In general, the ongoing spontaneous fluctuation is almost eliminated by averaging over realizations, but the response behavior manifests itself in the averaged signal. If the response of the network is weak, the network returns to its baseline ongoing activity quickly, whereas strong response causes a waveform of damped oscillation as illustrated in Fig. S2 (see also Fig. 3(c) in the maintext for the cases of a module in RN and MN).

**2.2.2 Measure the response sensitivity in the field models**

We can apply similar analysis in the field equations simulation. In the single-module field equation Eq. (S2.10), the stimulus is to raise the E, I membrane potential ($V_{E}$ and $V_{I}$) above the threshold to $-40mV$. Then, we measure the corresponding area index calculated from the average membrane potential of the module $V=0.8V_{E}+0.2V_{I}$. In the coupled version of the field equations Eq. (S2.6), we exert the above stimulus in a selected module $k$ and detect the response by the signal of this module as described above.


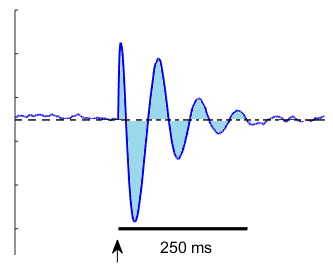


Fig. S2. The illustration of computing the response size. The colored area between the signal curve (the trial-average voltage or firing rate) and its pre-stimulus baseline value (dashed line) in 0~250ms after stimuli is used to measure the response size. Stimuli are applied at the time marked by the arrows. Refer to Fig. 3(c) for the cases of RN and MN in the network model.

**2.3 Mean-field reduction of neuronal network to coupled neural oscillators**

Now we derive the macroscopic field equations corresponding to the spiking network (S2.1). Denote $V_{E}^{k}=\left\langle V_{i}^{k} \right\rangle_{i\in k,E}$ , $V_{I}^{k}=\left\langle V_{i}^{k} \right\rangle_{i\in k,I}$,$\Phi_{E}^{k}=\left\langle{GE}_{i}^{k} \right\rangle_{i\in k,E or k,I}$ and $\Phi_{I}^{k}=\left\langle{GI}_{i}^{k} \right\rangle_{i\in k,E or k,I}$ as the average E , I voltage, E, I input conductance of the *k*-module. For external inputs of neuron $i$, we adopt a diffusion approximation that ${{GO}_{i}^{k}\left( t \right)\approx\tau}_{d}^{E}g_{e}\left( r_{in}+\sqrt{r_{in}} \xi_{i}^{k}\left( t \right) \right)$, with $\left\{ \xi_{i}^{k}\left( t \right) \right\}_{k,j}$being independent standard (with zero mean and unit variance) GWNs. Thus,$\left\langle{GO}_{i}^{k}\left( t \right) \right\rangle_{i\in k,\alpha}{\approx\tau}_{d}^{E}g_{e}\left( r_{in}+\sqrt{\frac{r_{in}}{N_{\alpha}}}\xi_{\alpha}^{k}\left( t \right) \right)$ , with $\left\{ \xi_{\alpha}^{k}\left( t \right) \right\}_{k,\alpha}$being independent GWNs. Taking the average and to the first equation of Eq. (S2.1), with the decoupling approximation: $\left\langle[{GE}_{i}^{k}+{GI}_{i}^{k}]V_{i}^{k} \right\rangle_{i\in k,\alpha}\approx\left\langle{GE}_{i}^{k}+{GI}_{i}^{k} \right\rangle_{i\in k,\alpha}\left\langle V_{i}^{k} \right\rangle_{i\in k,\alpha}$ we get

$\frac{dV_{\alpha}^{k}}{dt}=\frac{V_{rest}-V_{\alpha}^{k}}{\tau}+\left[ \tau_{d}^{E}g_{e}\left( r_{in}+\sqrt{\frac{r_{in}}{N_{\alpha}}} \xi_{\alpha}^{k}\left( t \right) \right)+\Phi_{E}^{k} \right]\left( V_{E}^{rev}-V_{\alpha}^{k} \right)+\Phi_{I}^{k}\left( V_{I}^{rev}-V_{\alpha}^{k} \right)$. (S2.2)

By the assumption [2], we know that the firing rate of the $\alpha$neurons in the *k*-th module can be approximated as

$Q_{\alpha}^{k}\left( t \right)=\left\langle\sum_{n} \delta\left( t-t_{j}^{k}\left( n \right) \right) \right\rangle_{j\in k,\alpha}=1/[1+exp(\frac{V_{th}-V_{\alpha}^{k}}{\sigma_{\alpha}^{k}}\frac{\pi}{\sqrt{3}})]$ . (S2.3)

Eq. (S2.3) essentially captures the sub and supra threshold microscopic dynamics of a spiking network, that is, $Q_{\alpha}^{k}(t)$ represents the proportion of $\alpha$ type neurons that spike between $t$ and $t+\Delta t$ ($\Delta t$ is an infinite small quantity) as well as the mean firing rate of $\alpha$ type neurons at time $t$ with unit per ms [2]. Here, $\sigma_{\alpha}^{k}$ are effective parameters to construct the voltage-dependent mean population firing rate. Note that this approximation scheme based only on the first-order statistics neglects several factors that affect the accurate firing rate, including higher order statistics, noise correlation and refractory time. Thus, $\sigma_{\alpha}^{k}$ does not have an analytical form and should be estimated numerically. A complete analytical approach for conductance-based integrate-and-fire neural network is still an open issue [3,4]. Furthermore, the quality of the scheme depends on suitable choices of effective parameters $\sigma_{\alpha}^{k}$ (see Eq. (S2.11) below).

Under mean-field approximation, we have $\left\langle\sum_{j\in\partial_{k,i}^{l,\alpha}} \sum_{n} \delta\left( t-t_{j}^{l}\left( n \right) \right) \right\rangle_{i\in k,E or k,I}=n_{\alpha}^{kl}Q_{\alpha}^{l}(t)$, where $n_{\alpha}^{kl}$ is the average number of $\alpha$ neighbors in the *l*-th module of a neuron in the *k*-th module. Thus, $n_{\alpha}^{kl}=p^{kl}N_{\alpha}$, where $p^{kl}$ is the connection probability from module $l$ to module $k$. In our network model, from Eq. (S1.5) and Eq. (S1.7), we have

$p^{kl}=\left\{ \begin{aligned} P_{intra}=P_{c}\left( 1+\left( N_{m}-1 \right)P_{r} \right), k=l \\ P_{inter}=P_{c}\left( 1-P_{r} \right), k\neq l \end{aligned} \right.$ . (S2.4)

Taking or to the second and third equations of Eq. (S2.1), we have

$\frac{d\Phi_{\alpha}^{k}}{dt}=-\frac{\Phi_{\alpha}^{k}}{\tau_{d}^{\alpha}}+g_{\alpha}N_{\alpha}P_{c}[\left( 1+\left( N_{m}-1 \right)P_{r} \right)Q_{\alpha}^{k}(t)+\sum_{l\neq k} \left( 1-P_{r} \right)Q_{\alpha}^{l}(t)$ . (S2.5)

Thus, the field equations of the whole MN are obtained as

$\left\{ \begin{aligned} \frac{dV_{\alpha}^{k}}{dt}=\frac{V_{rest}-V_{\alpha}^{k}}{\tau}+\left[ \tau_{d}^{E}g_{e}\left( r_{in}+\sqrt{\frac{r_{in}}{N_{\alpha}}} \xi_{\alpha}^{k}\left( t \right) \right)+\Phi_{E}^{k} \right]\left( V_{E}^{rev}-V_{\alpha}^{k} \right)+\Phi_{I}^{k}\left( V_{I}^{rev}-V_{\alpha}^{k} \right) \\ \frac{d\Phi_{\alpha}^{k}}{dt}=-\frac{\Phi_{\alpha}^{k}}{\tau_{d}^{\alpha}}+g_{\alpha}N_{\alpha}P_{c}[\frac{\left( 1+\left( N_{m}-1 \right)P_{r} \right)}{1+\exp\left( \frac{V_{th}-V_{\alpha}^{k}}{\sigma_{\alpha}^{k}}\frac{\pi}{\sqrt{3}} \right)}+\sum_{l\neq k} \frac{\left( 1-P_{r} \right)}{1+\exp\left( \frac{V_{th}-V_{\alpha}^{l}}{\sigma_{\alpha}^{l}}\frac{\pi}{\sqrt{3}} \right)},\alpha=E,I, k=1,\ldots,N_{m} \end{aligned} \right.$. (S2.6)

Since all modules are identical, we have $\sigma_{\alpha}^{k}=\sigma_{\alpha}$ for all *k*. Denote $X^{k}=\left( V_{E}^{k},V_{I}^{k},\Phi_{E}^{k},\Phi_{I}^{k} \right)^{T}$, we can write the field equations in the vector form as

$\frac{{dX}^{k}}{dt}=F\left( X^{k} \right)+\sum_{j=1}^{N_{m}} a_{kj}G(X^{j})$ , (S2.7)

with

$$F\left( X^{k} \right)=\{\frac{V_{rest}-V_{E}^{k}}{\tau}+\left[ \tau_{d}^{E}g_{e}\left( r_{in}+\sqrt{\frac{r_{in}}{N_{E}}} \xi_{E}^{k}\left( t \right) \right)+\Phi_{E}^{k} \right]\left( V_{E}^{rev}-V_{E}^{k} \right)+\Phi_{I}^{k}\left( V_{I}^{rev}-V_{E}^{k} \right),$$

$\frac{V_{rest}-V_{I}^{k}}{\tau}+\left[ \tau_{d}^{E}g_{e}\left( r_{in}+\sqrt{\frac{r_{in}}{N_{I}}} \xi_{I}^{k}\left( t \right) \right)+\Phi_{E}^{k} \right]\left( V_{E}^{rev}-V_{I}^{k} \right)+\Phi_{I}^{k}\left( V_{I}^{rev}-V_{I}^{k} \right),-\frac{\Phi_{E}^{k}}{\tau_{d}^{E}},-\frac{\Phi_{I}^{k}}{\tau_{d}^{I}}{\}}^{T}$ , (S2.8a)

$G\left( X^{k} \right)=\left\{ 0,0,\frac{g_{E}N_{E}P_{c}}{1+\exp\left( \frac{V_{th}-V_{E}^{k}}{\sigma_{E}}\frac{\pi}{\sqrt{3}} \right)},\frac{g_{I}N_{I}P_{c}}{1+\exp\left( \frac{V_{th}-V_{I}^{k}}{\sigma_{I}}\frac{\pi}{\sqrt{3}} \right)} \right\}^{T}$, (S2.8b)

$a_{kj}=\left\{ \begin{aligned} 1+\left( N_{m}-1 \right)P_{r}, k=j \\ 1-P_{r}, k\neq j \end{aligned} \right.$ . (S2.8c)

One should notice that the effective parameters $\sigma_{E},\sigma_{I}$ in Eq. (S2.6) should be determined by all other parameters (particular by rewiring probability $P_{r}$).

For example, if $\sigma_{E},\sigma_{I}$ are independent of $P_{r}$, then at the deterministic steady-state where $\dot{V_{\alpha}^{k}}=\dot{Q_{\alpha}^{k}}=\xi_{\alpha}^{k}=0$, one expect that $V_{\alpha}^{k}=V_{\alpha}^{l}$, $\Phi_{\alpha}^{k}=\Phi_{\alpha}^{l}$ , $Q_{\alpha}^{k}=Q_{\alpha}^{l}$ for all $k\neq l$ since the identity of different modules. Then, these steady values are solved by the algebraic equations

$\left\{ \begin{aligned} \frac{V_{rest}-V_{\alpha}^{ss}}{\tau}+\left[ \tau_{d}^{E}g_{e}r_{in}+\Phi_{E}^{ss} \right]\left( V_{E}^{rev}-V_{\alpha}^{ss} \right)+\Phi_{I}^{ss}\left( V_{I}^{rev}-V_{\alpha}^{ss} \right) \\ \Phi_{\alpha}^{ss}\left( t \right)={\tau_{d}^{\alpha}g}_{\alpha}N_{\alpha}P_{c}N_{m}/[1+\exp\left( \frac{V_{th}-V_{\alpha}^{ss}}{\sigma_{\alpha}}\frac{\pi}{\sqrt{3}} \right)] \end{aligned} \right.$ . (S2.9)

Thus, the steady-state firing rate $Q_{\alpha}^{ss}\left( t \right)=1/[1+exp(\frac{V_{th}-V_{\alpha}^{ss}}{\sigma_{\alpha}}\frac{\pi}{\sqrt{3}})]$ is independent of $P_{r}$ if $\sigma_{E},\sigma_{I}$ are independent of $P_{r}$, which cannot capture the effect of the firing rate reduction during rewiring (Fig. 2 in the main text).

**2.4 Field equations of a single module**

To understand the overall dynamic principle of the modular network, we can focus on analyzing the dynamics of single separate module. In the limit of $P_{r}\to1$ (all rewired), modules are almost separated. Let $P_{r}=1$ in Eq. (S2.6) and we get the field equations corresponding to one separate module with additional external excitatory inputs:

$\left\{ \begin{aligned} \frac{dV_{\alpha}}{dt}=\frac{V_{rest}-V_{\alpha}}{\tau}+\left[ \tau_{d}^{E}g_{e}\left( r_{in}+\sqrt{\frac{r_{in}}{N_{\alpha}}} \xi_{\alpha}\left( t \right) \right)+\Phi_{E} \right]\left( V_{E}^{rev}-V_{\alpha} \right)+\Phi_{I}\left( V_{I}^{rev}-V_{\alpha} \right)) \\ \frac{d\Phi_{\alpha}}{dt}=-\frac{\Phi_{\alpha}}{\tau_{d}^{\alpha}}+g_{\alpha}N_{\alpha}p_{c}/\left[ 1+\exp\left( \frac{V_{th}-V_{\alpha}}{\sigma_{\alpha}}\frac{\pi}{\sqrt{3}} \right) \right] , \alpha=E,I \end{aligned} \right.$ , (S2.10)

where $p_{c}=P_{c}N_{m}$ in the connection density of this module. From this we can know how the dynamic properties of a module depends on its effective connection density $p_{c}$. In our study here, the effective parameters $\sigma_{E},\sigma_{I}$ are estimated by

$\sigma_{\alpha}=\frac{V_{th}-V_{\alpha}}{ln({(Q_{\alpha}^{ss})}^{-1}-1)}\frac{\pi}{\sqrt{3}} , \alpha=E,I$, (S2.11)

where $V_{\alpha}^{ss}$ and $Q_{\alpha}^{ss}$ are the steady-state average membrane potential and firing rate of E, I neurons in the single separate module and they are estimated through numerical simulation of the module under different connection density $p_{c}$ (Fig. 4(a) bottom panel). Although qualitative prediction may not depend on the exact value of $\sigma_{\alpha}$, (see Fig. 6(b)), we adopt Eq. (S2.11) to achieve a better predictive outcome.

**Supplementary Notes III. Analysis of the Simplified Model with Correlated Inputs**

We measure the topological correlation in a RN by the ratio between the number of common neighbors and total number of distinct neighbors of a pair of neurons in the network, that is, $C_{topology}=\frac{(N{-2)P}_{c}P_{c}}{2(N-2)P_{c}-(N-2)P_{c}P_{c}}=\frac{P_{c}}{2-P_{c}}$. The spiking correlation (Fig. 4(a)) is measured by the average Person correlation of the spike trains (constructed with window 1 ms) of each neuron in the network.

The relationship between input correlation and response firing rate of the simplified model (Fig. 5(c)) can be obtained as follows. First, in the case without correlation ($C=0$), the synaptic signal for the target neuron is $s^{0}(t)=\sum_{i} {[s}_{i}\left( t \right)-\tau_{s}r]$, where $s_{i}(t)$ is the input of the *i*-th synapse at the time step $t$ and $\tau_{s}r$ is the mean of the signal such that $\left\langle s^{0}(t) \right\rangle=0$. We assume the input signal $s^{0}(t)$ is normally distributed (as in Fig. 4(c)) with the mean $E=0$ and the variance $\sigma^{2}\left( s^{0}(t) \right)=K\sigma^{2}\left( s_{i}(t) \right).$ For each synapse, as $s_{i}=0$ or 1 randomly, we have $\left\langle s_{i} \right\rangle=\left\langle s_{i}^{2} \right\rangle=\tau_{s}r$ and the variance is $\sigma^{2}\left( s_{i} \right)=\tau_{s}r-\left( \tau_{s}r \right)^{2}$. Thus, $\sigma^{2}\left( s^{0} \right)=K\left[ \tau_{s}r-\left( \tau_{s}r \right)^{2} \right]$. The output firing rate is determined by the probability that the input signal is above the threshold $\theta$. Using the error function the firing rate [5] is obtained as

$r_{out}=\frac{1-\mathrm{erf} \left( \frac{\theta}{\sqrt{2K\tau r_{s}(1-\tau_{s}r)}} \right)}{2\tau_{s}}$ , (S3.1)

as shown by the red dashed line ($C=0$) in the Fig. 5(c).

As the correlation of spikes is present in the $K$ input synapses ($C>0$) when a common spike train is added into all spike trains, the correlated spikes at the time *t* give the input signal $s^{c}\left( t \right)=K+\sum_{i} [s_{i}\left( t \right)-\tau_{s}\beta]$and its mean strength is $K,$ which can active the neuron at time *t* with probability 1 because $K\gg\theta$. As the rate of the correlated spikes is $\alpha=Cr$, the firing rate of the neuron is

$r_{out}^{c}\approx r_{out}+Cr$, (S3.2)

since the correlated spikes will always induce spiking of the neuron in this simplified model. Here in Eq. (S3.2), the $r_{out}$ is given by Eq. (S3.1) with $r$ replaced by $\beta=(1-C)r$.

**Supplementary references**

1. Wang S-J, Ouyang G and Guang J *et al.* Stochastic oscillation in self-organized critical states of small systems: Sensitive resting state in neural systems. *Phys Rev Lett* 2016;**116**:018101.

2. Liang J, Zhou T and Zhou C. Hopf Bifurcation in Mean Field Explains Critical Avalanches in Excitation-Inhibition Balanced Neuronal Networks: A Mechanism for Multiscale Variability. *Front Syst Neurosci* 2020;**14**:580011.

3. Brunel N and Wang X-J. Effects of neuromodulation in a cortical network model of object working memory dominated by recurrent inhibition. *J Comput Neurosci* 2001;**11**:63–85.

4. Renart A, Brunel N and Wang X-J. Mean-field theory of irregularly spiking neuronal populations and working memory in recurrent cortical networks. In Feng J (Ed) *Computational Neuroscience: A Comprehensive Approach* (pp. 431–490). London: CRC Press, 2004.

5. Kuhn A, Aertsen A and Rotter S. Higher-order statistics of input ensembles and the response of simple model neurons. *Neural Comput* 2003;**15**:67–101.

6. Beggs JM and Plenz D. Neuronal avalanches in neocortical circuits. *J Neurosci* 2003;**23**:11167–11177.

7. Shriki O, Alstott J and Carver F *et al.* Neuronal avalanches in the resting MEG of the human brain. *J Neurosci* 2013;**33**:7079–7090.

**Other supplementary figures**


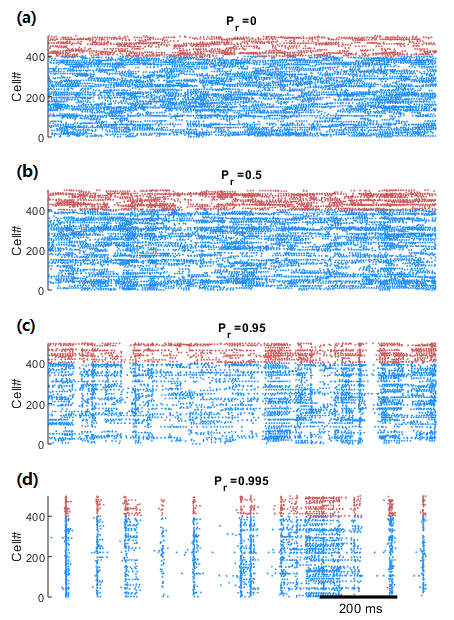


Fig. S3. The raster plot of spike times (blue, red for E, I cells) in a module selected from the whole network. From (a) to (d) the rewiring probability is $P_{r}=0, 0.5, 0.95, 0.995$ respectively. As $P_{r}$ grows, the dynamic mode transitions from asynchronous spiking to intermittent clustered spiking.


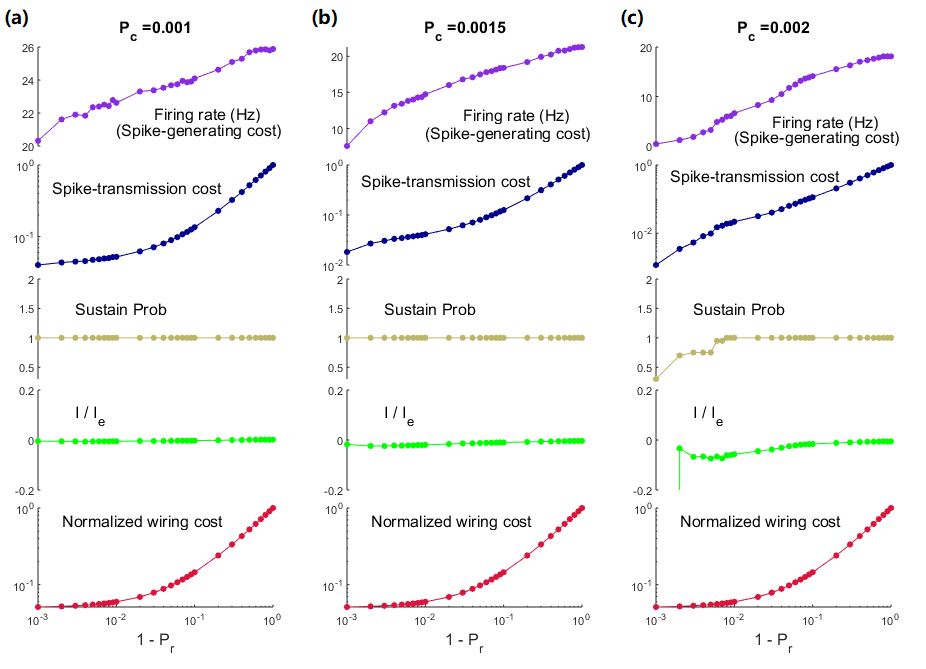


Fig. S4. Comparison of network properties during rewiring under different global density $P_{c}$. From top to bottom: the average firing rate; spike-transmission cost; sustained probability $P_{sus}$; current ratio $I/I_{e}$ and normalized wiring cost. (a) $P_{c}=0.001$. (b) $P_{c}=0.0015$. (c) $P_{c}=0.002$. Results in the main text are obtained by $P_{c}=0.0017$. Conclusions are the same when $P_{c}$ is around this value. However, MN (for $P_{r}\to1$) may not be able to self-sustain when $P_{c}$ is larger, as shown in (c). This can be understood from the self-sustain property of a single isolated module (Fig. 4(a)). However, since we do not employ synaptic scaling (i.e. let the synaptic strength decreases with neighbor numbers) in our model, the network cannot maintain E-I balance for too large $P_{c}$ (data not shown).


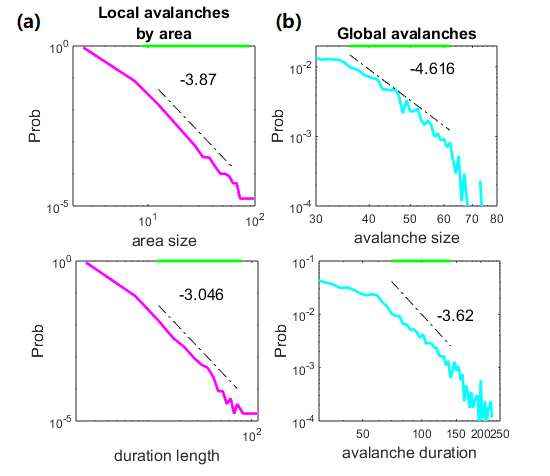


Fig. S5. Additional measurements of avalanches. In the main text, we measure avalanches from neuronal spikes (Fig. 3 and 4). Here, we present other measurements of avalanches using the average membrane potential associated with a threshold $V_{0}$. (a) Avalanches defined by the above-threshold-event of the average membrane potential $\left\langle V \right\rangle$ of a module, as used in [6]. The size of an avalanche is defined as the area below the curve $\left\langle V \right\rangle$ and above the threshold in the event. The duration of an avalanche is defined as the time length of the event. The size and duration probability distributions of avalanches defined in this way are shown ($P\left( S \right)\sim S^{-\tau}$, $\left( T \right)\sim T^{-\alpha}$ $\tau=3.87$, $\alpha=3.046$, with $p$ value $>0.1$). Measures are taken in a module of MN with $P_{r}=0.995$, $P_{c}=0.0017$. (b) Global avalanche between modules. The threshold events of each module is first defined when the average membrane potential of each module $\left\langle V^{k} \right\rangle$ reaches threshold $V_{0}$. Then the spread of threshold events in $N_{m}=100$ modules is analyzed with method similar to spiking data with a time bin $\Delta t=3.5ms$. This defines the mesoscopic avalanches as studied in MEG data [7]. Avalanche size and duration distribution are shown ($P\left( S \right)\sim S^{-\tau}$, $\left( T \right)\sim T^{-\alpha}$ $\tau=4.616$, $\alpha=3.62$, with $p$ value $>0.1$). Measures are taken in a module of MN with $P_{r}=0.99$, $P_{c}=0.0017$. In (a, b), a threshold $V_{0}=-60mV$ is used. The threshold is approximately the mean ($-66.7mV$) plus 2 times of the standard deviation ($2.9mV$) of $\left\langle V \right\rangle$, as in previous studies. The green lines on the top indicate the ranges of estimated power-law distributions. The results show that our model can exhibit critical avalanches in mesoscopic scale. However, the critical exponents are much smaller than those found in typical EEG/MEG studies [7], indicating that large activity cascades have much smaller impact at the global level. The difference may arise from the fact that the spatial scale of our model (a small cortical sheet) is different from the spatial scale of EEG/MEG measures (spanning almost the whole cortex). Thus, the properties of these mesoscopic avalanches deserve further study, for example, in terms of critical exponents and in association to the underlying spiking avalanches.


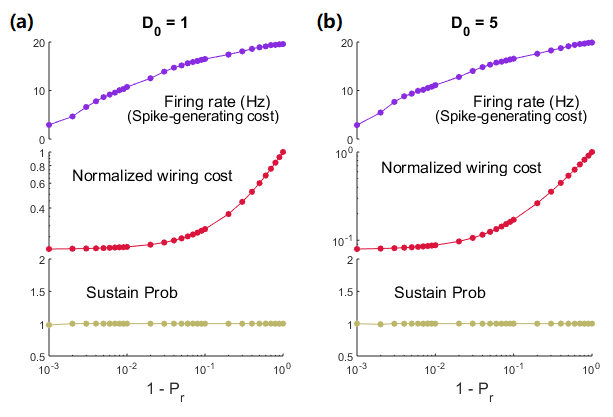


Fig. S6. Results of an extended model setting where the number of inter-modular links depends on the distance between modules. Here, the distance between two modules $k$ and $l$, denoted as $D_{kl}$, is defined as the distance between the center position of two modules and the number of inter-module links between two modules is proportional to $e^{-\frac{D_{kl}}{D_{0}}}$, where $D_{0}$ is a parameter which determines the characteristic length. From top to bottom: The firing rate, normalized wiring, and the sustained probability versus the rewiring probability are presented. $D_{0}=1$ and $D_{0}=5$ in (a) and (b) respectively. The network size is $N=50,000$ with density $P_{c}=0.0017.$


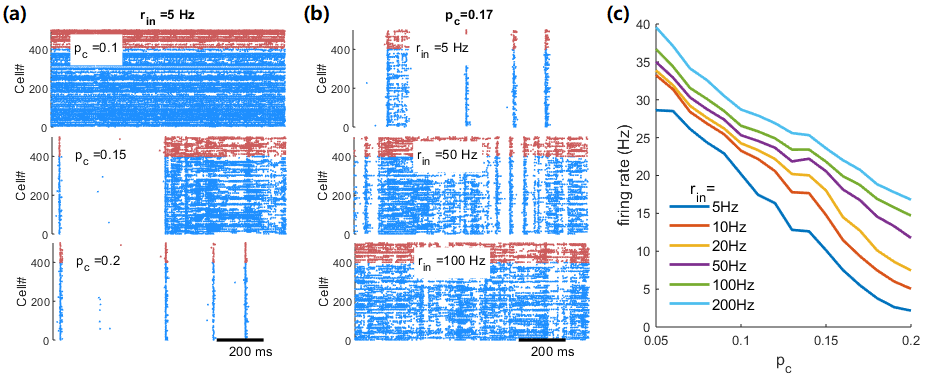


Fig. S7. Additional simulation of single separate module with different density $p_{c}$ and external input rate $r_{in}$. (a) The raster plot of spikes with $r_{in}=5$ Hz and connection densities $p_{c}=$ 0.10, 0.17, and 0.20 respectively. Under fixed input the dynamic transitions from asynchronous spiking to synchronous spiking with decreased rate. (b) The raster plot of spikes with density $p_{c}=$ 0.17 and $r_{in}$ = 5, 50, and 100 Hz respectively. Under fixed connection density, the dynamic transitions from synchronous spiking to asynchronous spiking as with increased input rates. (c) The firing rate with different density $p_{c}$ and input rate $r_{in}$. The firing rate of the network always decreases with the connection density under various input rates.


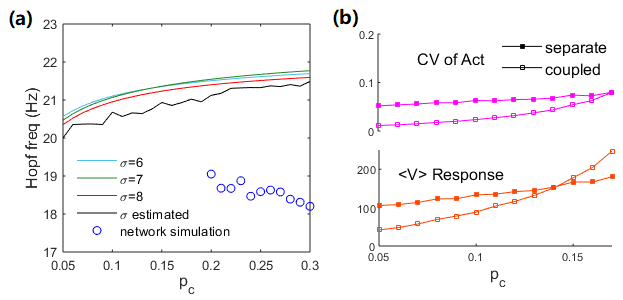


Fig. S8. Additional comparison of mean-field theory prediction. (a) The Hopf frequency, defined by imaginary part divided by 2π of the dominant eigenvalue of the fixed point, of the single-module field equation Eq. (2). Different curves are results by fixing $\sigma_{\alpha}=6,7,8$, and by ‘optimal’ $\sigma_{\alpha}$ given in Fig. 4(a). Blue circles are peak frequencies of $V_{E}$ oscillation from network simulation. Since the single module system approaches but has not reached the Hopf bifurcation point when increasing $p_{c}$ (Fig. 6(b)), together with the finite size effect from the small number of neurons in a module, the prediction of oscillatory frequency is not very precise with errors around a few Hz. (b) The comparison of the CV of activity and response size of membrane potential between 1) the single-module field equations (Eq. (2)) with different density $p_{c}$ (solid markers, results in Fig. 6(b)) and 2) the coupled-modules field equations (Eq. (5)) with corresponding modular density $p_{c}$ (hollow markers, transferred by results in Fig. 6(c) through $p_{c}=P_{c}(1+(N_{m}-1)P_{r})$).
